# Rolling in the Deep: Imaging Findings and Diagnostic Pearls in Gallstone Ileus

**DOI:** 10.1155/2020/1421753

**Published:** 2020-04-24

**Authors:** Carnevale Aldo, Motta Lorenzo, Labaj Olgerta, Cossu Alberto, Uccelli Licia, Giganti Melchiore

**Affiliations:** ^1^Department of Diagnostic and Interventional Radiology, University Radiology Unit, University Hospital of Ferrara, Ferrara, Italy; ^2^Department of Morphology, Surgery and Experimental Medicine, Section of Radiology, University of Ferrara, Ferrara, Italy; ^3^Department of Morphology, Surgery and Experimental Medicine, Section of Nuclear Medicine, University of Ferrara, Ferrara, Italy

## Abstract

Gallstone ileus is a dramatic complication of gallstone disease, uncommon but not exceptional in a busy emergency department. It represents a cause of mechanical intestinal obstruction, which predominantly occurs in elderly and frail patients; this contributes to the high morbidity and mortality rates associated with this condition. The modern radiologist is frequently asked to determine the cause of bowel obstruction and should be aware of the most pictorial features of this unusual disease. Broadly speaking, abdominal radiography and ultrasonography alone are limited in detecting the cause of bowel obstruction, but the sensitivity for the preoperative diagnosis of gallstone ileus may be improved by combining the findings obtained by both techniques. Computed tomography is the modality of choice for the diagnosis of this disease: it may accurately describe the number, size, and location of migrated gallstones and the exact site of bowel obstruction, providing a detailed preoperative planning. Magnetic resonance imaging may be used in selected cases for an exquisite anatomic definition of the fistulous communication.

## 1. Introduction

Firstly described in 1654 by Erasmus Bartholin, Danish physician and mathematician, gallstone ileus (GI) is an infrequent but dramatic complication of cholelithiasis; it constitutes an uncommon cause of mechanical bowel obstruction, but not exceptional in a busy emergency department. Despite medical advances over the last 350 years, it still represents a diagnostic and therapeutic dilemma, with a high mortality rate since it predominantly occurs in elderly, debilitated, and multimorbid patients. It follows that a prompt diagnosis is mandatory to avoid complications and allow an accurate preoperative planning. In the last two decades, some advances in the radiologic diagnosis of this condition by sonography, abdominal radiography, computed tomography, and sporadically magnetic resonance have been reported, offering new insights into this unusual entity.

GI represents a cause of mechanical intestinal obstruction resulting from gallstone migration and subsequent impaction into the bowel lumen. It is unusual among the causes of mechanical bowel obstruction accounting for 0.4–5% of cases [1], with a constant incidence of 30–35 cases out of 1,000,000 admissions over a 45-year period [2] and a mortality and morbidity rate of around 7–30% due to delayed diagnosis or misdiagnosis [3]. Indeed, this entity remains a significant diagnostic and treatment challenge.

GI usually occurs in debilitated patients with multiple comorbidities who had suffered from past episodes of calculous cholecystitis; elderly women are more frequently affected, with a female-to-male ratio of 6 : 1 [4] up to 16 : 1 [5] and an average age of 65−75 years.

**Figure 1:**
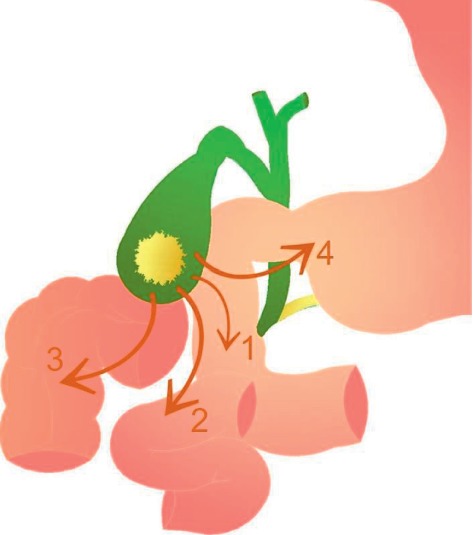
Schematic representation of the most common routes of biliary-enteric fistulation; in order of frequency: (1) cholecystoduodenal, (2) cholecystojejunal, (3) cholecystocolonic, and (4) cholecystogastric fistula.

The most common underlying cause of GI is represented by the presence of a biliary-enteric fistula (BEF). Cholecystoduodenal fistulas are the most frequent due to the proximity of the gallbladder to the duodenum, seen in 32.5–96.5% of cases (Table 1); cholecystojejunal, cholecystocolonic (5–25%), cholecystoduodenal-colonic (2.5%), and cholecystogastric fistulas [6] are as well described in the literature, but are rarer (Figure 1; Table 1). Although seldom associated with GI (0–13.4%) choledochoduodenal fistulas represent another possible route for gallstones to access the enteric lumen. This type of BEF is most commonly due to the dorsal erosion of a duodenal ulcer. The incidence of choledochoduodenal fistulas ranges from 4% up to 61% as reported by Petrowsky and Clavien in their 2006 systematic review [7]. Less frequently, in patients who have previously undergone ERCP and endoscopic Oddi's sphincterotomy, a large gallstone may migrate into the common bile duct, subsequently accessing the duodenum through the dilated major papilla.

GI onset is generally preceded by many episodes of silent or symptomatic biliary disease: recurrent and chronic inflammation of the gallbladder and surrounding tissues leads to adhesion formation, pressure necrosis, and wall erosion, with subsequent cholecystoenteric fistulation and gallstone migration.

The gallstone must be greater than 2–2.5 cm to cause bowel obstruction in the absence of a digestive pathology which can determine stenosis. Indeed, many smaller calculi may pass spontaneously through a normal gastrointestinal tract and may be excreted uneventfully in the stool [9]. The usual site of stone impaction is the distal ileum and the ileocecal valve (60%) since their lumens are narrow, but the possible locations of the lodged gallstone are diverse: jejunum (16%), stomach (14%), colon (4%), and duodenum (3.5%) [10]. While the gallstone causing obstruction is usually only one, it is important to be aware that additional calculi may be present in the bowel proximal to the site of occlusion, since they may represent the cause of GI recurrence after treatment. Some authors advocated that a cylindrical or faceted morphology of the calculus may be predictive of recurring GI [9]: this relies on the assumption that these shapes imply multiplicity of stones, which should alert the surgeon to the possibility of further stones in the remaining bowel.

From a clinical point of view, a large part of the patients will have a well-known history of prior acute cholecystitis or biliary symptoms. Most of the BEF are clinically silent. GI takes an insidious course: the presentation is typically nonspecific, with usually intermittent obstructive symptoms as the stone progresses aborally through the bowel, including nausea, vomiting, vague abdominal pain, and distension [11]. This intermittent onset has been referred to as a “tumbling” obstruction and may delay the final diagnosis. Eventually, signs of mechanical bowel obstruction may occur, including abdominal colicky pain and total constipation.

Bouveret's syndrome is a rare variant, seen in 1–3% of cases of GI [12]: a gallstone about 4.5 cm in diameter (larger than those producing the most common variants of GI) erodes the gallbladder into the duodenum, resulting in a cholecystoduodenal fistula (60%) and consequent gastric outlet obstruction, since the site of impaction occurs in the proximal duodenum or pylorus; less frequent locations are the gallbladder fossa and the stomach [1, 12].

Management of GI is controversial because of patient age and multiple comorbidities. Whilst open surgery has been the cornerstone of treatment, other approaches have been more recently employed, including endoscopy, laparoscopic surgery, and lithotripsy [13], although too few cases have been reported to formulate a conclusion about their effectiveness compared with standard methods. In selected cases, especially with elder and frail patients, endoscopy can be considered as a first-line noninvasive approach. Suitability of the endoscopic retrieval of a dislodged gallstone primarily depends on its size and final site of impaction along the enteric lumen. Endoscopically accessible large gallstones are amenable to various options of fragmentation techniques, including intra/extracorporeal lithotripsy, electrohydraulic lithotripsy, and mechanical lithotripsy. Furthermore, endoscopic repair of BEF is now a well-established possibility.

Among the surgical options, enterotomy with stone extraction alone appears to be more suitable: it is less invasive, with a low rate of complications [11]. In addition, spontaneous closure of the fistulous tract is observed in more than 50% of cases; however, recurrence has been reported in 2–5% of patients, due to a remaining gallstone entering a persistent fistula, or unseen further stones left in the bowel lumen during previous surgery [1]. Hence, alternative treatment options include enterolithotomy, cholecystectomy, and fistula repair as a one-stage procedure, or enterolithotomy with delayed cholecystectomy and fistula repair when the patient has recovered from the acute episode [13].

Here, we provide a comprehensive description of imaging findings in gallstone ileus, detailing possible pitfalls and pathognomonic relevance of the most pictorial features.

## 2. Conventional Abdominal Radiography

Plain abdominal radiography (XR) is routinely performed in emergency settings as a first-line imaging approach for investigating bowel obstruction; for decades, it had maintained the gold standard role in the diagnosis of GI.

**Figure 2:**
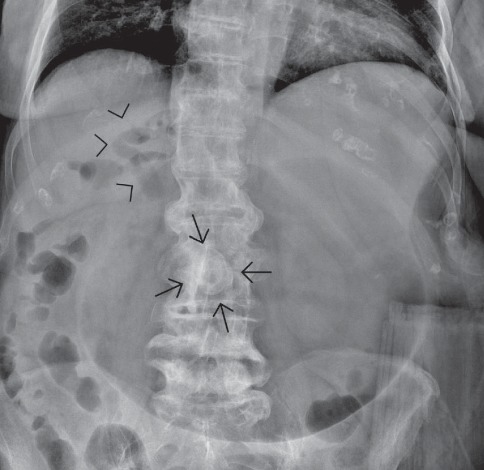
Rigler's triad in a supine abdominal radiograph. Note is made of the association of pneumobilia (arrowheads), a peripherally calcified stone (arrows) superimposed to the lumbar spine, and evident gastric overdistension; findings are consistent with gastric outlet obstruction due to a migrated large gallstone.

**Figure 3:**
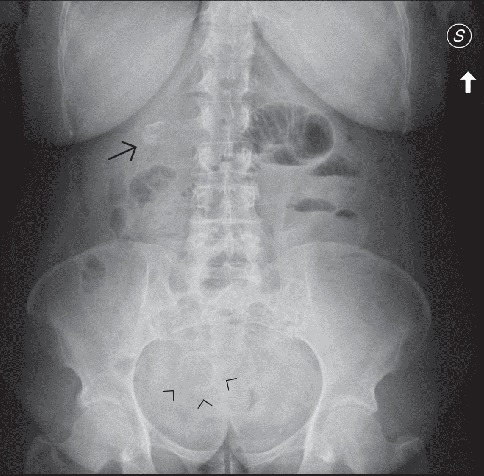
Upright abdominal radiograph shows the incomplete manifestation of the classical Rigler's triad in a patient with gallstone ileus. Note the paucity of intestinal gas, with dilated small bowel loops in the left upper abdomen and air-fluid levels. A stone with a thin peripheral calcified rim is visible (arrowheads), but pneumobilia is not detectable. Note curvilinear areas of increased opacity in the right upper quadrant, projecting into the gallbladder fossa (arrow).

The classic and pathognomonic imaging finding in GI consists in the association of air in the biliary tree (pneumobilia), ectopic radio-opaque stone, and small bowel obstruction (SBO), named after the radiology pioneer Leo Rigler as “Rigler's triad.” Originally described for abdominal XR, the modern radiologist is more likely to encounter its CT counterpart [14] (Figure 2). However, this characteristic association of findings has been shown to occur in less than 50% of patients, since it has been reported that up to a half of cases manifest two signs of the classical triad [15] (Figure 3).

At abdominal XR, the outstanding feature of SBO is distended small bowel ansae proximal to the site of obstruction with decompressed distal bowel; upright or left lateral decubitus films demonstrate multiple air-fluid levels. In proximal occlusions, as in the case of Bouveret's syndrome, the stomach may be dilated as well. Paucity of small-bowel gas, or “the gasless abdomen,” is another possible radiological presentation, due to fluid-filled dilated intestinal loops rather than endoluminal gas; this finding is more commonly seen in the supine radiograph, whereas the upright acquisition may demonstrate the “string of beads” sign, produced by small air bubbles entrapped in the intestinal folds between valvulae conniventes. The absence of small-bowel gas in the setting of SBO usually indicates a high-grade occlusion [16].

A wide range of sensitivity is reported in the literature for the diagnosis of GI on abdominal films. In a series of 27 patients with surgically confirmed GI [4], Rigler's triad was present in 4 cases with abdominal XR, thus providing the diagnosis in almost 15% of cases and in 21 cases with CT images. In 74% of cases, only signs of a mechanical ileus were identifiable on abdominal XR, therefore requiring further diagnostic investigation to clarify the cause of bowel obstruction.

In another series [17], the authors found that Rigler's triad could be observed in its complete manifestation in 36% of cases (5 out of 14 patients). The reason for this limited sensitivity may be due to obesity, superimposed bony structures, or fluid-filled bowel obscuring aberrantly located gallstones. Furthermore, only 20% of gallstones contain enough calcium to be identifiable on plain radiographs, as they are predominantly composed of cholesterol [18].

Of course, the radiologist evaluating an abdominal XR should always be aware of any change in gallstone position when compared to previous imaging studies, suggesting, in the absence of previous surgery, stone migration to anomalous sites.

The presence of an air collection in the gallbladder is another finding suggestive for GI, firstly reported by Balthazar and colleagues [19] who identified a typical association of two adjacent small air-fluid levels in the right upper abdominal quadrant representing the duodenal bulb adjacent to the air and fluid-filled gallbladder.

However, it should be borne in mind that pneumobilia may result from prior surgical or endoscopic biliary manipulation, and therefore, the clinical picture should be taken into consideration when evaluating this radiographic sign in order to avoid misdiagnosis [20].

**Figure 4:**
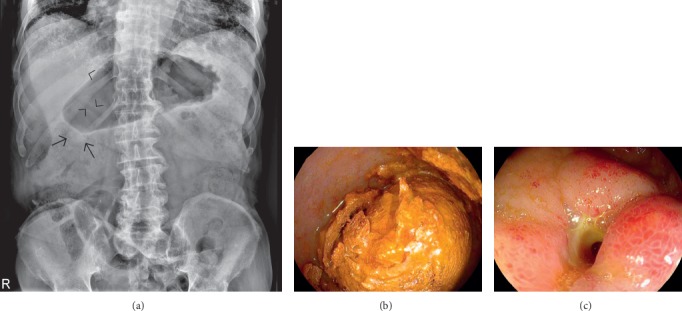
Supine abdominal radiograph (a) in a case of Bouveret's syndrome demonstrates pneumobilia (arrowheads) and a dilated stomach. An ectopic slightly radiopaque stone in the right upper abdominal quadrant was originally missed (arrows). Esophagogastroduodenoscopy in the same patient reveals an impacted large gallstone in the duodenal bulb (b). The fistulous stoma is well visualized in (c).

In 64 reported cases of Bouveret's syndrome [21], the most frequent findings observed at abdominal XR were pneumobilia (39%), a calcified mass in the gallbladder region or in the right upper abdominal quadrant (38%), dilated stomach (23%), or small bowel loops (14%) (Figure 4).

## 3. Ultrasonography

Abdominal XRs are limited in detecting the cause of bowel mechanical obstruction, since most gallstones are not sufficiently opaque to be depicted. Abdominal ultrasonography (US), on the other hand, is the imaging method of choice for diagnosing gallstone disease [18], due to high sensitivity and accuracy (>95%), relatively low-cost, noninvasiveness, and lack of ionizing radiation, with the possibility of a bedside examination. In the diagnosis of GI, US may actually provide a useful adjunct to abdominal XR. However, in cases of bowel obstruction, it could be technically challenging due to patient discomfort and gaseous/fluid distension of bowel loops.

We do not routinely perform abdominal US in an adult patient suspected of having SBO, but in many institutions, the increased use of ultrasounds in the initial assessment of patients with abdominal pain has made point-of-care US relevant also to the diagnosis and characterisation of SBO [22].

A varying association of GI sonographic imaging findings has been described in the literature, including pneumobilia or air in the gallbladder fossa, detection of aberrantly located gallstone, bowel loop distension and wall swelling, and peritoneal fluid [5].

**Figure 5:**
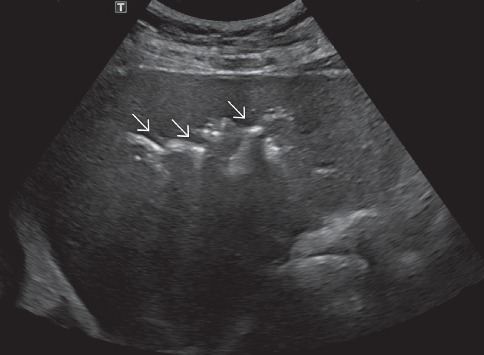
Abdominal ultrasound demonstrates the appearance of pneumobilia: bright echogenic foci in linear configuration (arrows) with posterior acoustic shadowing and reverberation artifacts.

Pneumobilia is excellently detected at US, with greater sensitivity than XR [5]: reflective linear echoes, more centrally located than portal pneumatosis, casting posterior acoustic shadowing and reverberation artifacts will be seen (Figure 5).

In a series of 23 patients [5], the authors found that US could improve sensitivity for definitive preoperative diagnosis of GI (74%) by combining abdominal XR and US findings, unlike plain film alone that demonstrated a lower sensitivity (17%). In the same work, Rigler's triad was identified by plain abdominal film in two patients (9%) and by US in 16 patients (69%). However, other works reported a lower accuracy for the US diagnosis of GI, with Rigler's triad being observed in almost 11% of cases [4].

## 4. Computed Tomography

With a sensitivity of 90–93%, specificity of 100%, and accuracy of 99% [17], computed tomography (CT) is considered the main diagnostic imaging technique in investigating bowel obstruction, or even any other cause of acute abdomen. It plays an essential role in the early detection of GI, enabling an accurate preoperative assessment and a faster surgical planning.

**Figure 6:**
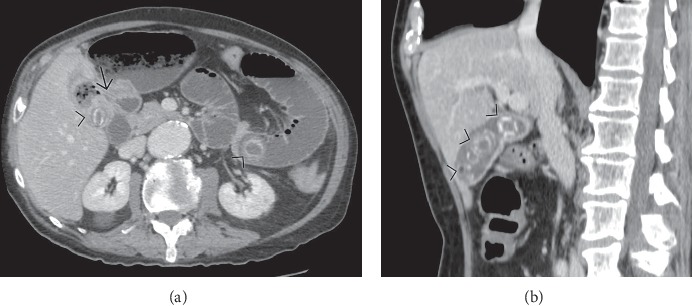
Contrast-enhanced computed tomography (CT) in a patient with gallstone ileus: two peripherally calcified stones (arrowheads in (a)) are present, the first visible in the gallbladder and the latter enlodged in the jejunum; the fistulous tract between the gallbladder and the duodenum is well visualized (arrow). A sagittal reformatted CT image obtained a few years before shows the gallbladder lumen filled with multiples stones (b).

The most common signs associated with GI were previously defined by Yu et al. [17] and include SBO; rim-calcified or total-calcified ectopic gallstone; and abnormal gallbladder with complete air collection, presence of air-fluid level, or fluid accumulation with irregular wall. The fistulous connection may be directly identified as well, but in a small percentage of patients [9] (Figure 6).

When a bowel obstruction is suspected, the standard protocol for CT scanning provides the use of intravenous (IV) contrast material with a portal venous phase acquisition [16]. IV contrast media enhances the bowel wall as well as the other abdominal viscera, which may allow exclusion of alternative diagnoses in patients with acute abdomen. In addition, contrast-enhanced CT is particularly beneficial to assess for the detection of bowel oedema, inflammation, and ischemia as possible complications of gallstone impaction. Dilated intestinal loops proximal to the site of stone impaction, recognized as the transition point from distended to collapsed distal bowel, air-fluid levels, and “string of beads” sign are related to mechanical occlusion [16]. Free fluid, portal venous, and mural gas are late signs more frequently associated with ischemic damage.

Even nonenhanced CT acquisition may be valuable in case of patients with a known history of contrast allergy or with renal impairment, leading in some cases to a better detection of ectopic rim-calcified gallstones which would be missed as part of bowel wall or alimentary content [17], or to better definition of alternative diagnoses or characterisation of unexpected collateral findings. Therefore, an unenhanced acquisition is usually obtained as well in our emergency department.

**Figure 7:**
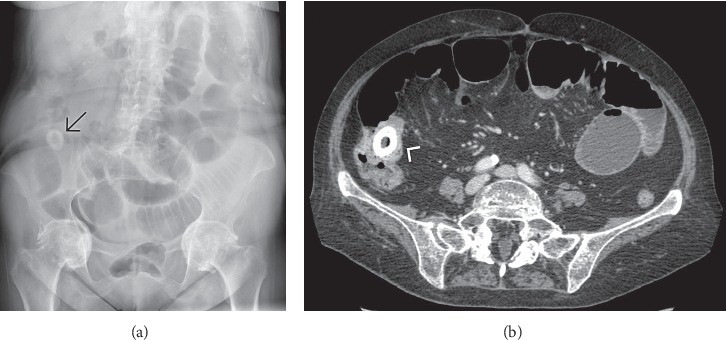
Supine abdominal radiograph (a) and contrast-enhanced computed tomography (b) in a case of gallstone ileus with a large calcified stone lodged at the level of the ileocecal valve, the most common site of bowel obstruction.

Moreover, even if sometimes the detection of gallstones may be difficult depending on their composition, CT scan with multiplanar reformatted images can exactly locate the migration site of the ectopic stones (Figure 7), establishing their size and number, and the consequent level of bowel obstruction. It is crucial to correctly report the presence of additional stones in the jejunum so that the surgeon can accurately search for them during the operation to prevent recurrence [9].

The correct evaluation of gallstone size is relevant too, since also those smaller than 2 cm may be dangerous as they may become larger by accretion while descending the intestinal canal and thus produce reflex spasm or volvulus [9].

**Figure 8:**
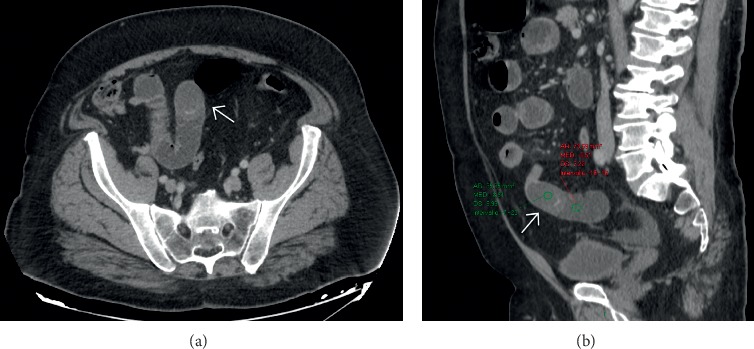
Contrast-enhanced computed tomography in a surgically proven case of gallstone ileus, in which the presence of an isodense gallstone (white arrows in (a) and (b)) in the jejunum was overlooked. Sagittal reformatted image (b) demonstrates a focal widening of the jejunum just before the transition point; this mass effect is associated with the presence of different attenuation values compared with the fluid-filled proximal bowel.

Nonetheless, the accurate definition of the size of the gallstone lodged in the bowel lumen could sometimes be challenging, due to various components of the gallstone itself with different densities. In their work, Gan and colleagues [12] defined subtle but useful imaging clues to better delineate the actual dimensions of the stone: on CT, the comparison of the fluid attenuation outside its calcified component (the “soft-tissue-density sign”), the presence of a linear air collection around its surface in a dependent fashion (the air-crescent sign), and a faint radiolucency due to air or fat in the stone outside the calcific rim could reveal the true size of the gallstone. Moreover, a focal widening of the bowel just before the transition point may suggest the presence of a noncalcific mass inside its lumen producing the obstruction (Figure 8).

**Figure 9:**
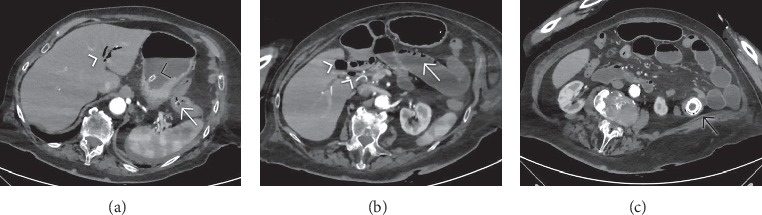
Contrast-enhanced computed tomography in the same patient than Figure 2, after nasogastric tube positioning (tip with black arrowhead in (a) demonstrates the CT counterpart of classical Rigler's triad: pneumobilia (white arrowheads in (a) and (b)), a peripherally calcified stone (black arrow in (c)) impacted in the proximal ileum lumen, and concomitant signs of mechanical intestinal obstruction, including dilated and partially fluid-filled small bowel loops proximal to the site of obstruction and decompressed distal bowel; air-fluid levels and “string of beads” sign (white arrow in (b)) are visible as well. An additional smaller calculus, not visible at abdominal radiography, is seen in (a) (white arrow).

**Figure 10:**
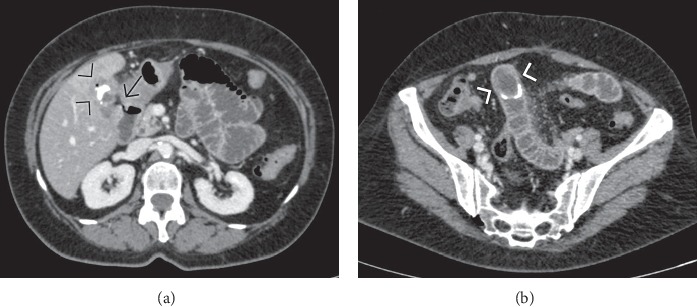
Contrast-enhanced computed tomography in the same patient than Figure 3 allows the depiction of a cholecystoduodenal fistula (arrow) with a linear air collection entrapped in the fistulous communication. Please note in (a) a small air bubble in the gallbladder lumen not detectable at abdominal radiograph, and rim calcification of the gallbladder wall (porcelain gallbladder; arrowheads). Dilated fluid-filled small bowel loops and decompressed distal small bowel consistent with obstruction are evident in (a) and (b). The point of transition corresponds to an isoattenuation intraluminal filling defect with a thin peripheral calcified rim.

Even if abdominal XR may demonstrate diagnostic features of GI, namely, Rigler's triad, CT is more accurate in detecting such pathognomonic association of findings [4, 9] (Figures 9 and 10).

**Figure 11:**
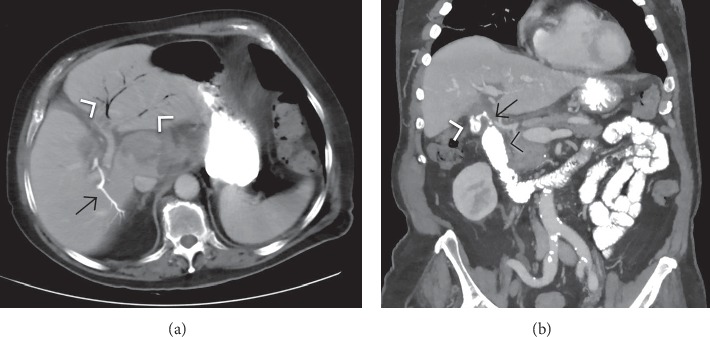
Contrast-enhanced computed tomography, 5-mm thickness multiplanar reconstruction, portal phase, demonstrates in (a) both pneumobilia (arrowheads) and opacification of the right lobe biliary branches (arrow) after oral contrast media administration; the fistula (arrow) between the gallbladder filled with contrast material (white arrowhead) and the duodenum (black arrowhead) is delineated in the coronal plane reconstruction shown in (b).

The use of hyperattenuating oral contrast agent is finalized to better visualize the anatomy of the bowel. In the case of GI, it may help to detect the fistulous path extending between the gallbladder and the gastrointestinal tract, visualized as contrast accumulation within the gallbladder [17, 23] (Figure 11).

However, many institutions have omitted the routine administration of high-attenuation oral contrast media in SBO investigation for several reasons: patients are frequently nauseated and may vomit, with a risk of aspiration; the bowel just proximal to the transition point could be rarely opacified in case of a high-grade obstruction; and the low-attenuation content (fluid and gas) within the bowel lumen usually provides excellent contrast with the wall, which may be obscured on the contrary by high-attenuation oral contrast material [16]. Furthermore, in the specific case of GI, the presence of hyperdense contrast material may mask the contours of calcified stones in the bowel lumen. For all the aforementioned reasons, in our institution, oral high-attenuation contrast material is not administered in patients suspected of having SBO.

**Figure 12:**
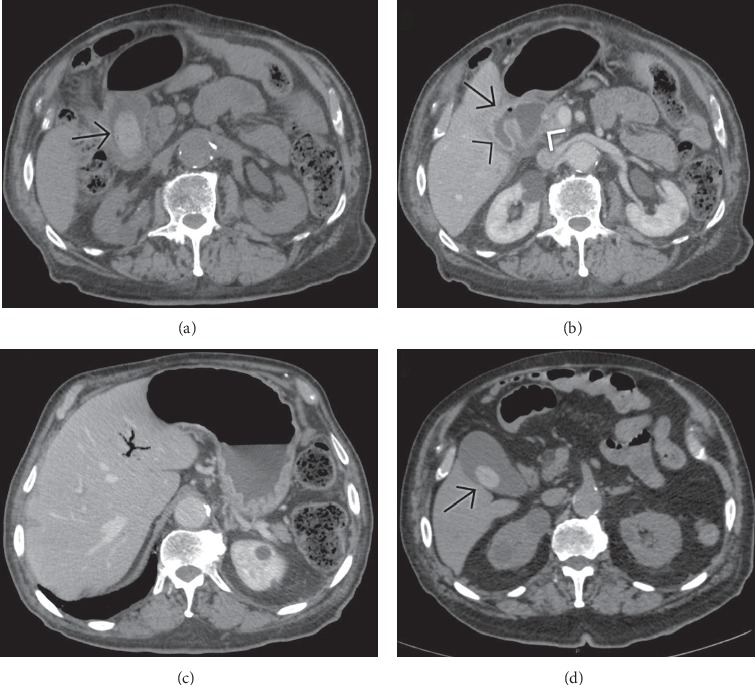
Unenhanced computed tomography in the same case of Bouveret's syndrome than Figure 4(a) shows a large slightly hyperattenuating filling defect (arrow) in the duodenal bulb. A fistula between the gallbladder (black arrowhead) and the duodenum (white arrowhead) is evident in (b) after intravenous contrast media administration. The association of ectopic gallstone visualization, pneumobilia (seen in (c)), and signs of intestinal obstruction (gastric overdistension visible in (c)) constitute Rigler's triad. In a previous unenhanced computed tomography (d), a smaller hyperattenuating calculus was detectable in the gallbladder: this finding represents a clue to the diagnosis of gallstone ileus.

With respect to Bouveret's syndrome, reviewing 40 CT studies of this rare GI variant, Cappell and Davis [2] found that pneumobilia was observed in only 60% of cases, ectopic gallstones in 42%, and evidence of gastric or duodenal obstruction in 33% (Figure 12).

Possible mimicker of gallstone ileus on CT imaging is represented by a small group of other nonstrangulating cause of bowel obstruction; among them is worth mentioning that primary enterolith of the upper GI tract, highly attenuating bezoars or other foreign body, may closely resemble the CT appearance of a migrated stone. Primary enterolithiasis is an uncommon cause of small bowel obstruction. It is generally accepted that the production of enteroliths is favoured by states of intestinal dysmotility, especially in cases of duodenal or jejunal diverticulosis. Mostly composed of choleic acid and other bile metabolism products, these stones tend to have similar CT features of gallstone, being usually calcified and round-shaped [24]. Absence of biliary-enteric fistula and normal appearance of the gallbladder may suggest the diagnosis. Historically classified according to the involved/ingested material, bezoars may show a wide spectrum of imaging appearance, primarily depending on their composition and age. Some bezoar, as in persimmon seeds overconsumption (phytobezoar), may present as an endoluminal gas-containing mass-like structure, with numerous hyperdense small components (seeds) interspersed within the mass itself. Clinical context and absence of biliary pathology may lead to the correct diagnosis.

## 5. Magnetic Resonance Imaging

Magnetic resonance cholangiopancreatography (MRCP) represents the imaging modality of choice in investigating biliary tree pathologies since it provides fine anatomical details, allowing the detection of small calculi and clarifying the differential diagnosis with other biliary conditions. Despite its high sensitivity, MRCP is not routinely performed in acutely ill patients most of all because of longer time of acquisition and insufficient panoramicity. This technique has a sensitivity of 97.7% for gallstone detection, higher than abdominal XR (40–70%), CT scan (90–93%), and even than US (95%), especially in cases of isoattenuating and radiolucent stones that can be missed at abdominal XR and CT, or even in cases of very small ones (<3 mm) [25, 26].

**Figure 13:**
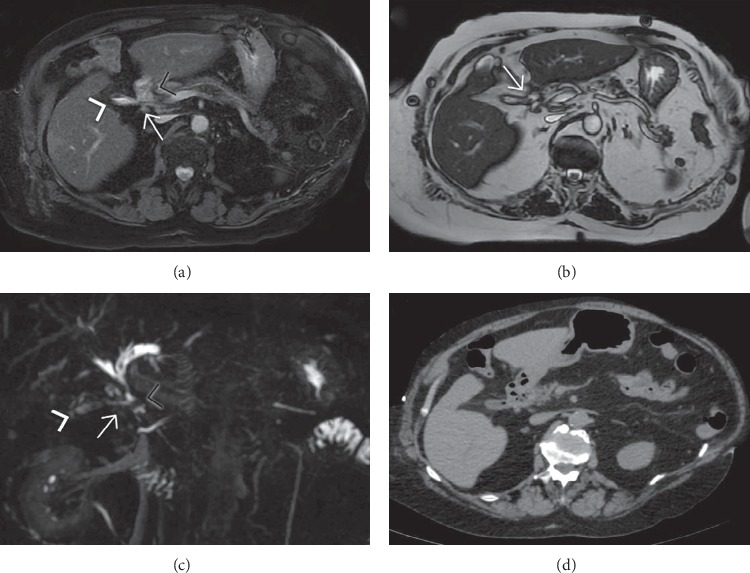
Magnetic resonance imaging, fat-suppressed FIESTA axial sequence (a), SSFSE T2-weighted axial sequence (b), MIP-reformatted 3D-MRCP sequence (c), and unenhanced computed tomography of a patient with iodinate contrast media allergy treated for gallstone ileus with enterolithotomy. The fistulous communication (arrow in (a), (b), and (c)) between the gallbladder (white arrowhead in (a), (b), and (c)) and the duodenum (black arrowhead in (a), (b), and (c)) is readily visible in the MR images due to the presence of a small amount of fluid in its collapsed lumen. The CT study (d) could not provide an accurate fistula anatomy required by the abdominal surgeon to decide for a possible fistula repair.

MRCP application in GI diagnosis and characterisation is almost anecdotal in the literature. This technique may arguably be useful in selected cases of GI with the potential to further improve diagnostic yield for noncalcified calculi due to the excellent discrimination of fluid from gallstones, the latter appearing on T2 weighted images as signal voids in the context of the high-signal fluid. Moreover, if sufficient fluid is present in a collapsed cholecystoenteric fistulous tract, it may be delineated on MR images [2]: in their work, Liang and colleagues [25] concluded that MR was more sensitive than CT in detecting and accurately describing the fistula anatomy (100%) (Figure 13).

Furthermore, MRCP together with CT may play a potential role in the management of chronic stones inside the gallbladder, helping in estimating the tendency to fistulation towards the bowel [25]: gallstone size greater than 2 cm, loss or blurring of the fat plane between the gallbladder and duodenum, patients over 70 years old, and previous common bile duct endoscopic basket lithotomy have been identified as possible risk factors for bilioenteric fistula forming.

## 6. Conclusion

Abdominal XR and US are first-level imaging modalities widely performed in the emergency setting to investigate patients with acute abdomen but are quite insensitive in the diagnosis of GI. Nowadays, CT is the imaging modality of choice for this disease, as well as in identifying alternative causes for patient symptoms: it provides a detailed description of the multiplicity, precise size, and location of the migrated gallstones, thus guiding the surgeon towards an accurate preoperative planning to prevent complications like recurrence due to unseen additional calculi remaining in the proximal bowel. In selected cases, MRI might be used as a potential adjunct in precisely defining the fistula anatomy.

## Figures and Tables

**Table 1:** Biliary-enteric fistulas absolute frequency and relative frequency in GI.

	BEF absolute frequency (%)	BEF frequency in GI (%)
Cholecystoduodenal	18–76	32.5–96.5
Cholecystogastric	2.5–5	0–13.3
Cholecystojejunal	0–14.7	0–2.5
Cholecystocolonic	9–17	0–10.9
Choledochoduodenal	4–61	0–13.4

Data are expressed in percentage ranges according to reference [2, 6–8].
